# The Effects of Stress on Cognitive Aging, Physiology and Emotion (ESCAPE) Project

**DOI:** 10.1186/s12888-015-0497-7

**Published:** 2015-07-03

**Authors:** Stacey B. Scott, Jennifer E. Graham-Engeland, Christopher G. Engeland, Joshua M. Smyth, David M. Almeida, Mindy J. Katz, Richard B. Lipton, Jacqueline A. Mogle, Elizabeth Munoz, Nilam Ram, Martin J. Sliwinski

**Affiliations:** School of Aging Studies, University of South Florida, Tampa, USA; Department of Biobehavioral Health, Pennsylvania State University, Pennsylvania, USA; Department of Biobehavioral Health and College of Nursing, Pennsylvania State University, Pennsylvania, USA; Departments of Biobehavioral Health and Medicine, Pennsylvania State University and Hershey Medical Center, Pennsylvania, USA; Department of Human Development and Family Studies and Center for Healthy Aging, Pennsylvania State University, Pennsylvania, USA; Department of Neurology, Albert Einstein College of Medicine, Bronx, USA; Department of Neurology, Department of Psychiatry and Behavioral Sciences, Department of Epidemiology and Population Health, Albert Einstein College of Medicine, Bronx, USA; Center for Healthy Aging and College of Nursing, Pennsylvania State University, University Park, Pennsylvania, PA 16802 USA; Department of Human Development and Family Studies and Center for Healthy Aging, Pennsylvania State University, Pennsylvania, USA; Department of Human Development and Family Studies, Pennsylvania State University, Pennsylvania, USA; Department of Human Development and Family Studies and Center for Healthy Aging, Pennsylvania State University, Pennsylvania, USA

**Keywords:** Stress, Cognition, Aging, Inflammation, Ecological momentary assessment, Unconstructive repetitive thought, Cytokines, Cortisol, Burst measurement design

## Abstract

**Background:**

Despite evidence that psychological stress is an important risk factor for age-related cognitive loss, little research has directly evaluated psychological and physiological mediators of the relationship between stressful experiences and cognitive function. A key objective of the ESCAPE (Effects of Stress on Cognitive Aging, Physiology, and Emotion) project is to evaluate whether engaging in stress-related unconstructive repetitive thought (URT) is a pathway through which stressful experiences negatively affect cognitive health over the short- and long-term. Over the short-term, we hypothesize that engaging in URT will deplete attentional resources and result in worse cognitive performance in daily life. Over the long-term, we expect that the effects of chronic stress, from repeated exposure to stressors and regular engagement in URT, will be apparent in dysregulated hypothalamic-pituitary-adrenal (HPA) axis function and inflammation. Over time, stress-related physiological dysregulation will result in accelerated cognitive decline.

**Methods/Design:**

This study utilizes a prospective longitudinal measurement-burst design. A systematic probability sample of participants aged 25 to 65 is recruited from residents of the Bronx, NY. Consenting participants complete a baseline assessment and follow-up waves at 9, 18, and 27 months post-baseline. At each wave, participants complete a 14 day measurement burst of brief surveys and cognitive assessments delivered via study smartphones during daily life. Participants provide saliva samples four times each day for five days during the measurement burst and fasting blood samples at the end of each burst from which cortisol and dehydroepiandrosterone sulfate (DHEAS), circulating inflammatory markers, and stimulated inflammatory responses to lipopolysaccharide in whole blood are determined.

**Discussion:**

This study takes a multi-pronged approach to assessing stress (i.e., early adversity, chronic strains, major events, daily hassles), psychological mediators (e.g., URT), biological mechanisms (i.e., HPA function, inflammation) and outcomes across different time-scales (i.e., momentary cognitive performance, cognitive decline across years). The systematic probability sample is locally representative and can be compared with national norms on key markers of health and well-being. The findings will improve our understanding of how environmental, psychological, and physiological stress-related influences accumulate to affect cognitive health and identify potential targets (e.g., URT, inflammation) for prevention and intervention promoting cognitive health.

## Background

More than 5.4 million US adults over the age of 70 have cognitive impairment without dementia [1]; another 4.7 million have a diagnosis of Alzheimer’s dementia [2]. With a large and increasing proportion of the population over the age of 65, the costs of healthcare, long-term care, and hospice related to dementia and cognitive impairment are projected to reach $1.1 trillion dollars in 2050 [3]. Identification of modifiable risk factors in midlife, prior to the development of cognitive impairment in old age, represents a critical challenge to improving quality of life and controlling health care costs. Psychological stress is an important risk factor in this regard, because it relates to a broad range of aging-related health outcomes [4–8] and because it represents a target for prevention and intervention strategies.

Psychological stress can affect cognitive function in the short-term (e.g., as when an individual’s thoughts are occupied with an argument that happened earlier in the day resulting in reduced ability to pay attention to, keep track of, or remember steps in the task at hand) as well as over the long-term (e.g., as when those who experience chronic stress show accelerated cognitive decline compared to their less-stressed peers of the same age). Research supports these patterns - in the short-term, minor daily stressors can produce transient effects on cognition by reducing the amount of attentional resources available for information processing [9, 10]. During times when individuals appraise events and situations in their lives to be stressful, they allocate cognitive resources to coping with these demands [11, 12]; this in turn limits available resources and results in lower cognitive performance than during non-stress times. Daily stress has also been associated with short-term increases in inflammation as well as in negative mood; both inflammation and negative mood are associated with fatigue and may further explain reductions in attentional resources that can occur with acute stress [13]. Over the long-term, chronic life stress has been consistently associated with poorer cognitive function [8, 14], accelerated cognitive decline [5, 15], and increased incidence of dementia [16]. One explanation for these long-term effects is that individuals who experience chronic stress are at increased risk for biological ‘wear and tear’ (i.e., allostatic load; [17, 18]), that results in both dysregulated endocrine function and pro-inflammatory effects [19–22] which can impair the neural structure and function underlying cognitive performance [23–25].

Despite this body of evidence linking both daily and chronic stress to cognitive function, few studies have directly examined mediators to explain how stressful experiences affect cognitive function. Stress theorists have hypothesized that unconstructive repetitive thinking, which encompasses a range of related concepts such as worry and rumination, plays an important role in conveying the effects of stress on somatic and mental health [26, 27]. The Effects of Stress on Cognitive Aging, Physiology, and Emotions (ESCAPE) project consists of two National Institute on Aging R01-funded studies (R01 AG039409, R01 AG042595). Below, we describe the conceptual framework that guides the ESCAPE project.

### Conceptual framework

**Fig. 1:**
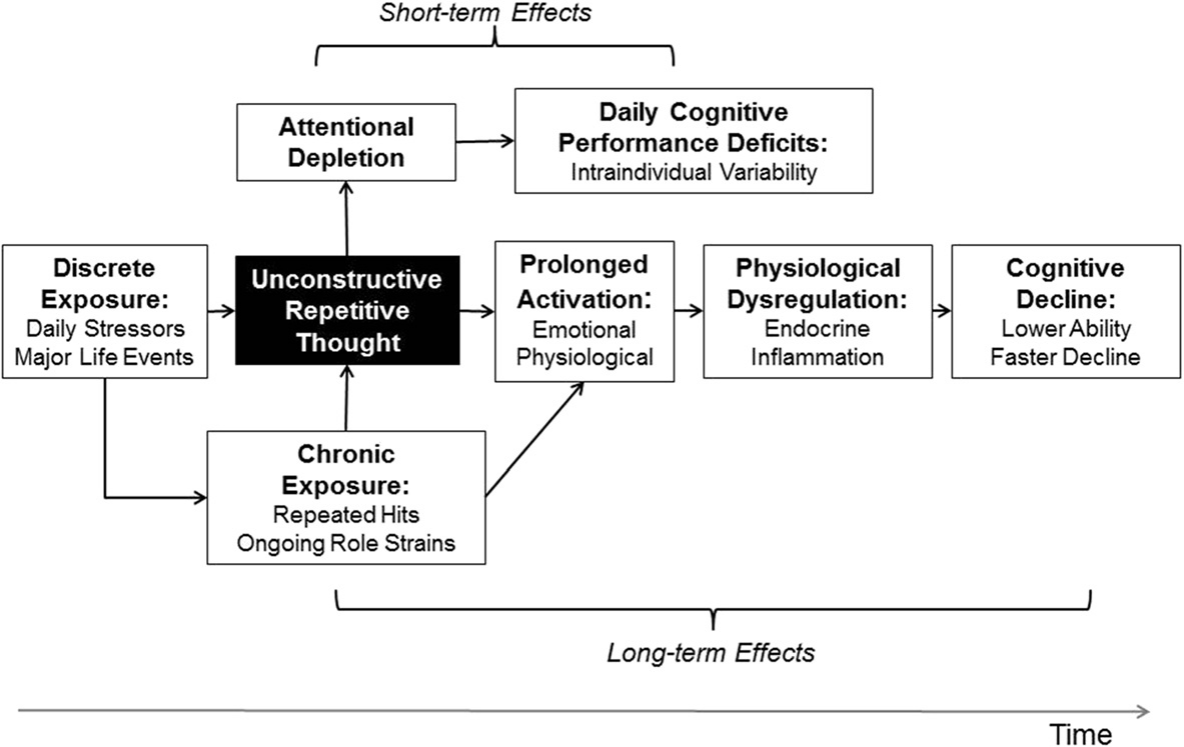
Model Guiding the Design and Hypotheses of the ESCAPE Study

Repetitive thought refers to the process of “thinking attentively, repetitively or frequently about one’s self and one’s world” [28]. Although some forms of repetitive thinking, such as reflection and problem solving, can be adaptive, other forms of repetitive thinking can be maladaptive. Unhelpful approaches to thinking about problematic situations or events have been broadly labeled unconstructive repetitive thinking (URT). In this model, URT subsumes related constructs such as rumination, worry, perseverative cognition, and cognitive interference [29]. Stressful experiences increase the likelihood of engaging in URT, and in turn, recurrent thinking about problematic situations and events can amplify, extend, and reactivate emotional and physiological components of the acute stress response, even after cessation of the eliciting stressor [14, 30]. The model in Fig. 1 guides the hypotheses and design of the ESCAPE project. URT is proposed as a psychological mechanism that prolongs physiological and emotional responses to daily and chronic stress which, in turn, can have short- and long-term negative consequences for cognitive function. Stress-related dysregulations of physiological systems, particularly of hypothalamic-pituitary-adrenal (HPA) axis function and inflammation, are proposed as physiological mechanisms that result in reduced cognitive function over the long-term.

The ESCAPE project will test whether URT accounts for the short- and long-term effects of stress on cognitive performance directly by depleting attentional resources, and indirectly by contributing to physiological dysregulation. First, we will evaluate whether stress-related URT produces short term negative effects on cognitive function by reducing the amount of attentional resources available for information processing (labeled as short-term effects in Fig. 1). For example, we will test whether recently engaging in URT explains why individuals have worse cognitive performance when they have recently experienced a stressor, compared to times when no stressors have occurred. Additionally, we will test whether engaging in URT predicts worse cognitive performance even when a stressor has not recently occurred. We expect these effects to be observed in the short term, over the course of days and across moments within a day. Second, we will test the mediational pathways through URT and physiological dysregulation (labeled long-term effects in Fig. 1). Specifically, we will examine whether the tendency to engage in URT helps explain how, over the long-term, chronic stress contributes to physiological dysregulation and, in turn, cognitive decline. We will evaluate whether, over months and years, stress-related alterations of HPA-axis function and inflammation mediate connections between URT and declines in cognitive performance.

## Methods/Design

### Project overview

The ESCAPE project is an ongoing longitudinal *measurement burst* study of a systematically recruited sample of racially and economically diverse 25–65 year olds residing in the Bronx, New York, U.S. Measurement burst designs consist of repeated sequences of closely spaced measurements [31–33] that provide multiple time scales that allow for examination of processes unfolding within individuals over short time periods (e.g., minutes, hours, days) and how these processes change over longer time intervals (e.g., months, years; see [34]). The ESCAPE project consists of 4 longitudinal waves of data collection focused on cognition, stress, URT, and physiological variables. During each of these waves, participants complete a 14 day measurement burst. Each measurement burst consists of brief surveys and cognitive assessments administered on study-provided smartphones that participants complete up to 5 times per day as they go about their daily lives. This design will produce intensive data on each participant (i.e., 4 waves with 14 days of 5 momentary observations per day resulting in up to 280 momentary observations) across a 3 year period. With these data, we will be able to disentangle the time-ordering of the relationships outlined in the model in Fig. 1. Related to our short-term predictions, we will use the measurement burst data to examine the extent to which stressor occurrence, followed by engagement in URT, results in lower cognitive performance later in the day. Related to our long-term predictions, we will use the wave-to-wave longitudinal data to test whether the tendency to engage in URT predicts elevations in systemic inflammation and steeper decline in cognitive performance across three years.

### Study site & participants

The Albert Einstein College of Medicine of Yeshiva University ethical review board approved the ESCAPE study protocol. Eligible participants are 25–65 years of age, ambulatory, fluent in English, free of visual impairment that would interfere with operating the study smartphone, and residing in Bronx County, New York. Exclusion criteria include inability to answer smartphone surveys throughout the day (e.g., due to work requirements or other commitments that would not allow participation in the study). The target sample size is 320 participants, which is based on power calculations required to identify small to medium sized effects for the primary study hypotheses.

The ESCAPE sample was selected to provide strong tests of the model in Fig. 1. First, the effects of stress on cognitive aging are theorized to accumulate across time and prior to old age; we therefore sampled young and middle-aged adults in order to capture this wear-and-tear as it occurs and affects cognitive functioning, prior to the full-blown development of cognitive impairment at older ages. Second, because both stress exposure and cognitive performance have been linked to socio-economic status, ESCAPE uses systematic probability sampling to recruit a racially and economically diverse sample of 25–65 year olds from Co-Op City, a Bronx, New York housing cooperative of approximately 60,000 residents.

### Procedure

The ESCAPE protocol involves 4 waves of data collection across a 3 year period. Each wave, participants complete paper-based dispositional surveys, a lab visit, an EMA phase with smartphone surveys and cognitive tasks and saliva assessments, and a wrap-up visit with blood draws and other health assessments. Payments were paid upon completion of each wave. Payments for participation were based upon compliance rates with the study protocol. Participants received a maximum of $160 each wave if all lab visits, blood draws, paper and pencil surveys are completed as well as 80 % compliance was met on completion of burst phase smartphone surveys and cognitive assessments. Below, we elaborate on recruitment and each phase of data collection.

#### Recruitment

A sampling frame was developed from New York City Registered Voter Lists (RVL) obtained from the Board of Elections. Based on their age, individuals on the RVL were categorized into 10-year age bins and assigned to sampling blocks consisting of 450 potential participants in each bin. Individuals in a sampling block were sent letters of introduction, explaining the project’s goals and how the recipient was identified. Within two weeks of sending recruitment letters, a follow up telephone call was conducted to establish rapport, identify exclusions, and enroll participants in the study.

#### Baseline and follow-up surveys

Following the recruitment phone call, consenting participants were mailed a baseline survey to complete at home. This survey contained demographic questions as well as detailed questionnaires about the major concentrations of this study – stress, URT, physical and mental health, and psychosocial factors that may moderate other observed relationships (see Measures section below and Table 1 for a full description). At follow-up waves, participants received similar survey packets in the mail and completed them prior to returning the packets at their lab visit.

**Table 1:** Measures

Construct	Domain	Description	Measures
Stress	Chronic	*Role and environmental stress, neighborhood security, discrimination*	Wheaton Chronic Stress (WCS) Scale, Lifetime Discrimination Scale, Neighborhood Safety and Violence Scale [70–72]
	Major events	*Lifetime & recent exposure to major life events and trauma (e.g., bereavement, abuse)*	Lifetime Adversity and Recent Life Event subscales of WCS [70]
	Subjective	*Perceived stressfulness of life over last month*	Perceived Stress Scale [73]
	Daily events	*Exposure to daily hassles and stressors (e.g., arguments, deadlines, illness)*	Adapted from Daily Inventory of Stressful Experiences [74]
URT	Event-related	*Recurring thoughts about major stressor*	Impact of Events Scale-Revised [75]
	Dispositional	*Tendency to ruminate or reflect*	Rumination-Reflection Questionnaire [38]; White Bear Suppression Inventory [37]
	Momentary	*Experiencing recurring, unwanted, negative thoughts*	Momentary Thought Questionnaire
Cognition	Laboratory	*Working memory*	Operation-span, Counting-span, Backward letter span
		*episodic memory*	AVLT, Paired-associates, Card Memory Task
		*processing speed*	Letter, Number and Symbol matching
		*fluid and crystallized intelligence*	Ravens Progressive Matrices; Vocabulary
	Ambulatory	*Working memory (verbal, spatial), processing speed*	See Fig. 2
Biomarkers	Laboratory	*Basal inflammatory markers*	Plasma cytokines: IL-1β, IL-2, IL-4, IL-5, IL-6, IL-8, IL-10, TNF-α, IFN-γ, GM-CSF; Plasma CRP
	Ambulatory	*LPS-stimulated cytokines*	Plasma cytokines: IL-1β, IL-6, IL-8, IL-10, TNF-α, GM-CSF
		*Stress-related hormones*	Salivary cortisol sampled 4 times per day for the first 5 days of each burst
			Plasma cortisol; Plasma DHEAS
Physical & Mental Health	Objective	*Metabolic risk indicators*	Lipid profiles; insulin; glucose
	Subjective	*Physical functioning, medical history, medication use, anxiety, depression, mood over last month, life satisfaction*	PROMIS Physical Function Short Form-A, PROMIS Emotional Distress – Anxiety – Short Form 7-A, PROMIS Depression Short From 8-A, Positive and Negative Affect Schedule, Satisfaction with Life Scale [64, 76, 77]
Risk & Protective Factors	Risk	*Neuroticism, social isolation, childhood adversity, anger*	International Personality Item Pool (IPIP) Neuroticism subscale, PROMIS Social Isolation – Short Form-6a, Childhood Adversity subscale from WCS, State-Trait Anger Expression Inventory [70, 78, 79]
	Protective	*Conscientiousness, social support, positive events, optimism, adult temperament, mindfulness*	IPIP Conscientiousness subscale, PROMIS Emotional, Informational, and Instrumental Social Support – Short Forms-6a, Pleasant Events Schedule, Life Orientation Test, Adult Temperament Questionnaire, Five Facet Mindfulness Questionnaire [64, 78, 80–83]
Demographics	Person	*Age, race, ethnicity, gender*	Demographic questionnaire
	Context	*income, work status, marital status, education*	

#### Lab visits

At the initial lab visit for each wave, participants received training in the use of custom-configured smart phones for the measurement burst protocol and complete lab-based cognitive tasks.

#### Training session

In the training session, participants learned how to operate the study smartphone, practiced the smartphone survey questions and cognitive tests (described in Measures section) with the research assistant, and asked questions about the smartphones and protocol. Participants also received training in the use of the saliva collection kits (described in Measures section). The research assistant explained the schedule of data collection and showed participants the Help Line number on the back of the phone which they could call if they encountered problems with the smartphone or questions about the data collection.

#### Lab-based cognitive assessments

Lab-based cognitive assessments occurred at each wave and consisted of face-to-face and computer-based tests of working memory, episodic memory, and processing speed (see Measures section and Table 1).

#### EMA practice phase

In the first wave of the study, participants completed a 2-day EMA practice phase after their initial lab visit and training. This served as a period in which participants became familiar with the study protocol. During the EMA practice phase, data was collected through smartphones and salivary assessments, described below. Participants returned to the lab at the end of the EMA practice phase and their compliance with the study protocol was calculated. Those participants who completed 80 % or more of morning, beeped, and bedtime smartphone surveys were invited into the burst phase of the study. Participants who did not meet compliance criteria were thanked and provided payment commensurate with their participation.

#### Smartphone surveys

Each morning, participants completed a brief smartphone survey about their previous night’s sleep and their expectations regarding how pleasant and stressful the day ahead will be. The smartphone produced an audible alert (“beep”) 5 times during the day, signaling participants to complete a survey about their recent stressors, current activities and emotions, and recent thoughts. At the completion of each of these surveys, the smartphone launched three brief cognitive tasks for the participant to complete. Beeps were scheduled in order to sample the entire waking day, with quasi-random timing to ensure that participants do not anticipate the beeps. At the end of each day, participants completed a separate bedtime survey in which they reported on their physical symptoms. The smartphone data was stored on the phone and data were sent in an encrypted format to a secure server upon completion of each survey.

#### Saliva assessments

Saliva was self-collected four times daily during the first 5 days of this period. Upon waking each morning, participants placed a synthetic Salivette (Sarstedt Inc., Newton NC) into their mouth and were instructed to chew on it gently until saturated. They then placed the Salivette into a pre-labeled collection tube and stored it in their refrigerator or freezer until their return visit to the clinic. Saliva samples were collected again in an identical manner 15 min and 30 min post-waking, and at bedtime. The specific times of sample collection were recorded by the participant on a form that was returned to investigators at the end of the burst.

#### Burst phase

Participants who met compliance criteria in the EMA practice phase were invited to follow the same protocol (i.e., 1 morning, 5 beeped, 1 evening survey each day) for 14 consecutive days. For the first 5 days of the burst phase, participants completed saliva assessments as in the EMA practice phase (i.e., upon waking, 15 min post-waking, 30 min post-waking, bedtime). Research staff made follow-up calls after the first day of smartphone surveys and again at the end of week 1 to motivate compliance and address any questions that arise during the burst assessments.

#### Wrap-up visit

Participants returned to the lab for a brief wrap-up visit after the 14-day burst phase and to return the study smartphones and saliva samples. During this visit a qualified phlebotomist obtained 12-h fasting blood samples, which were brought to the Institute for Clinical and Translational Research (ICTR) at Albert Einstein College of Medicine. The ICTR distributed samples for immediate analysis to the appropriate laboratories; samples were processed and aliquots of whole blood, plasma, and serum were stored at −70 °C for future analysis. Physical assessments were also obtained at this visit.

#### Longitudinal assessment schedule

There are 3 waves of follow-up data collection scheduled for 9, 18, and 27 months post baseline. As the study is currently in progress and has rolling recruitment, participants are currently in different waves. To limit participant burden, participants do not complete the EMA practice phase in follow-up waves. The first two lab visits were condensed into a single visit in which participants completed a brief refresher training on smartphone use and saliva collection, and they completed lab-based cognitive assessments. Prior to this visit, they completed a survey packet that included questionnaires to assess physical health, mental health, stress, personality, and psychosocial factors (e.g., social support). Participants next completed the burst phase, and then the wrap-up visit.

### Measures

The measures and timing of participants’ assessments are summarized in Table 1. Below, we describe the central domains assessed.

#### Demographic

In addition to conventional demographic variables describing stable characteristics of individuals (e.g., age, race, ethnicity, gender) assessed at baseline, we conducted follow-up assessments of aspects of individuals’ life context that may vary over time (e.g., income, work, marital status) that may vary over time and affect exposure and response to stressors.

#### Stress

The term “stress” describes a multicomponent process that, as measured in ESCAPE, has four major components that are aligned with different time-scales. Specific measures used to assess each component are shown in Table 1. *Chronic stress* describes sources of stress (e.g., caregiving, dangerous neighborhood; [35, 36]), many of which do not have clear onset or endings. *Major life events* are infrequent, dramatic stressors, and include events such as divorce, widowhood. *Subjective stress* describes general appraisal of the state of feeling distressed or overwhelmed over the last month. *Daily events* are possibly more frequent and/or recurrent stressors that are less dramatic and include events such as arguments and deadlines. Table 1 describes the specific instruments employed in ESCAPE to measure these components of stress (i.e., chronic stress, major life events, subjective stress, and daily events).

#### Unconstructive Repetitive Thoughts (URT)

We also assessed several different ways in which URT may play a role in individuals’ responses to major life events, chronic stressors, and daily hassles. Table 1 describes the specific measures used to assess both dispositional and state (i.e., momentary) URT. First, *dispositional URT* describes tendencies for some individuals to engage in recurrent negative thoughts more than others, both in general [37, 38] as well as in response to specific sources of major life stress [39]. We expect that dispositional measures (i.e., WBSI, RRQ, IES-R) will operate through influencing the frequency and intensity of momentary experiences of URT, both in response to a stressor as well as in the absence of external triggers. S*tate URT* was assessed using 4 items developed for this study. These items ask individuals to use a continuous visual analogue scale (a slider) to rate the thoughts they had experienced in the 5 min prior to completing the smartphone survey in terms of their overall valence (unpleasant—pleasant), negative self-focus (“Were you thinking about personal problems or worries?”), and controllability (“Were you experiencing a train of thought that you couldn’t get out of your head?” and “Were you preoccupied with thoughts of something about to happen or that might happen in the future?”).

#### Cognition

We used two complementary approaches to assessing cognitive function. First, lab-based cognition was assessed during visits to the research clinic by a trained technician in a controlled testing environment. Second, ambulatory cognition was assessed repeatedly via smartphones in naturalistic settings as people go about their daily activities. We expect that ambulatory assessments will provide a more ecologically valid characterization of individuals’ cognitive functioning that will complement and extend traditional lab-based assessments.

Lab-based cognitive assessments included *fluid intelligence and crystallized intelligence*, assessed by the Raven’s Progressive Matrices [40], and the WRAT/Vocabulary [41], in addition to working memory, episodic memory, and processing speed. *Working memory* (WM) reflects a person’s ability to manipulate and maintain information in an active form. Three standardized tasks were used to measure WM in the lab: counting span, operation span [42, 43], and the backwards letter-span task [44]. *Episodic memory* was measured using the Auditory Verbal Learning Test (AVLT) [45] and a word-number paired associates learning task [46]. The “Card-memory Task” (CMT) required participants to learn the location of 6 playing cards that appear on a computer monitor, and recall those locations after completing a visual distractor task. Participants completed two choice reaction time tasks as measures of *processing speed*. In two separate tasks, participants completed two strings of characters (letters or numbers) as quickly as possible. Strings were 3, 6, or 9 characters in length to provide a variety of difficulty levels for analysis. The Symbol-symbol comparison task required participants to decide as quickly as possible whether a target pair of non-verbalizable symbols matches any of a set of five comparison pairs. Stimuli were randomly generated for each of cognitive tasks to avoid stimulus specific learning effects across repeated measures.

**Fig. 2:**
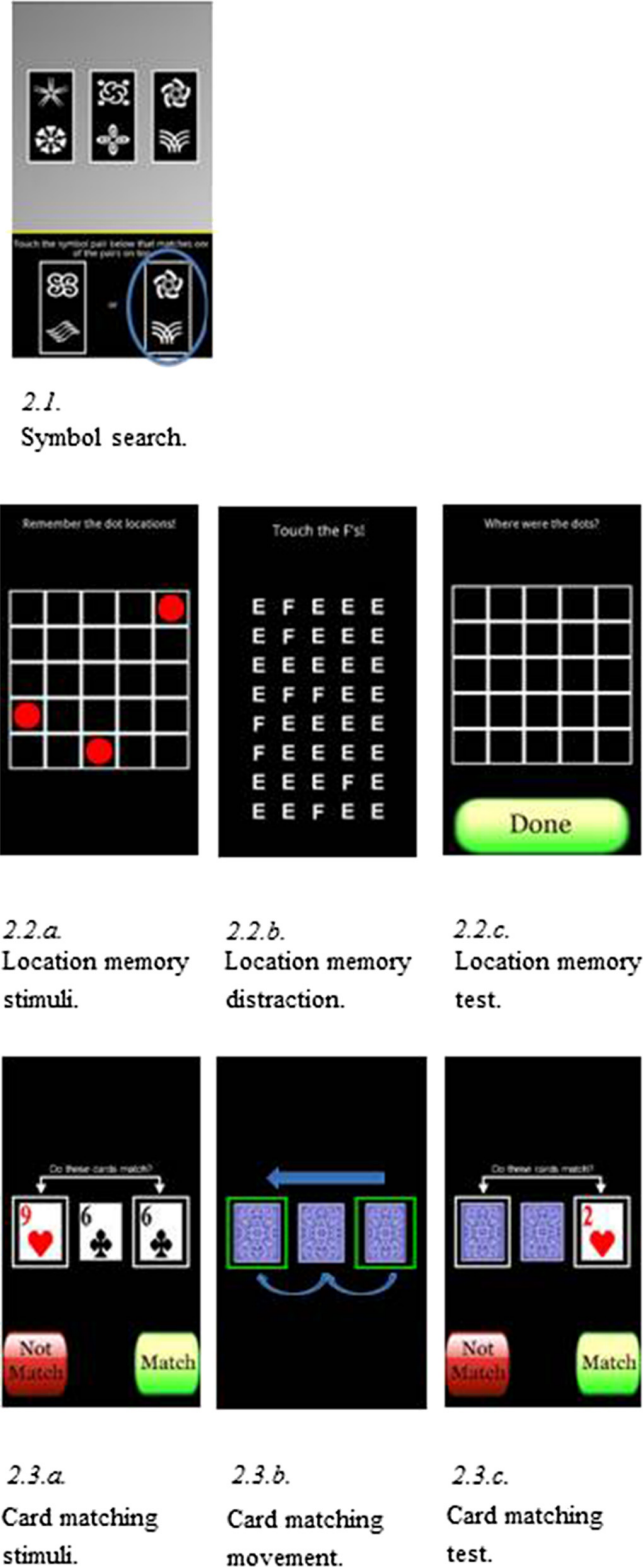
Smartphone Tasks for Assessing Ambulatory Cognition

Ambulatory cognition was assessed on the smartphone by three tasks presented after each beeped survey in the following fixed order: processing speed, spatial working memory, and verbal working memory. Screen shots are provided in Fig. 2. The smartphone *processing speed* tasks required participants to compare three symbol pairs at the top of the screen and with two symbol pairs at the bottom of the screen and decide as quickly as possible which of the two pairs presented at the bottom of the screen is among the pairs at the top of the screen (see Fig. 2.1). The location memory task assessed s*patial working memory* and required participants to memorize the location of three red dots that appear on a 5 x 5 grid for 3 s (see Fig. 2.2a). After a visual distractor exercise of 8-s (see Fig. 2.2b), participants then recalled the locations of the 3 dots (see Fig. 2.2c). The v*erbal working memory* was adapted from the columnized n-back paradigm [47], and included a 0-back and 2-back condition. Participants saw a series of 3 standard playing cards (see Fig. 2.3a) slide from one box on the right of the screen to the second box on left of the screen (see Fig. 2.3b). They completed a 0-back and 2-back variation of the standard n-back task. In the 0-back, cards face up and participants were asked to determine whether the cards in the two boxes match. In the 2-back variation, the previous two cards face down (see Fig. 2.3c). Each assessment consisted of 16 symbol comparison trials, 2 dot-memory trials, and 12 2-back trials.

**Fig. 3:**
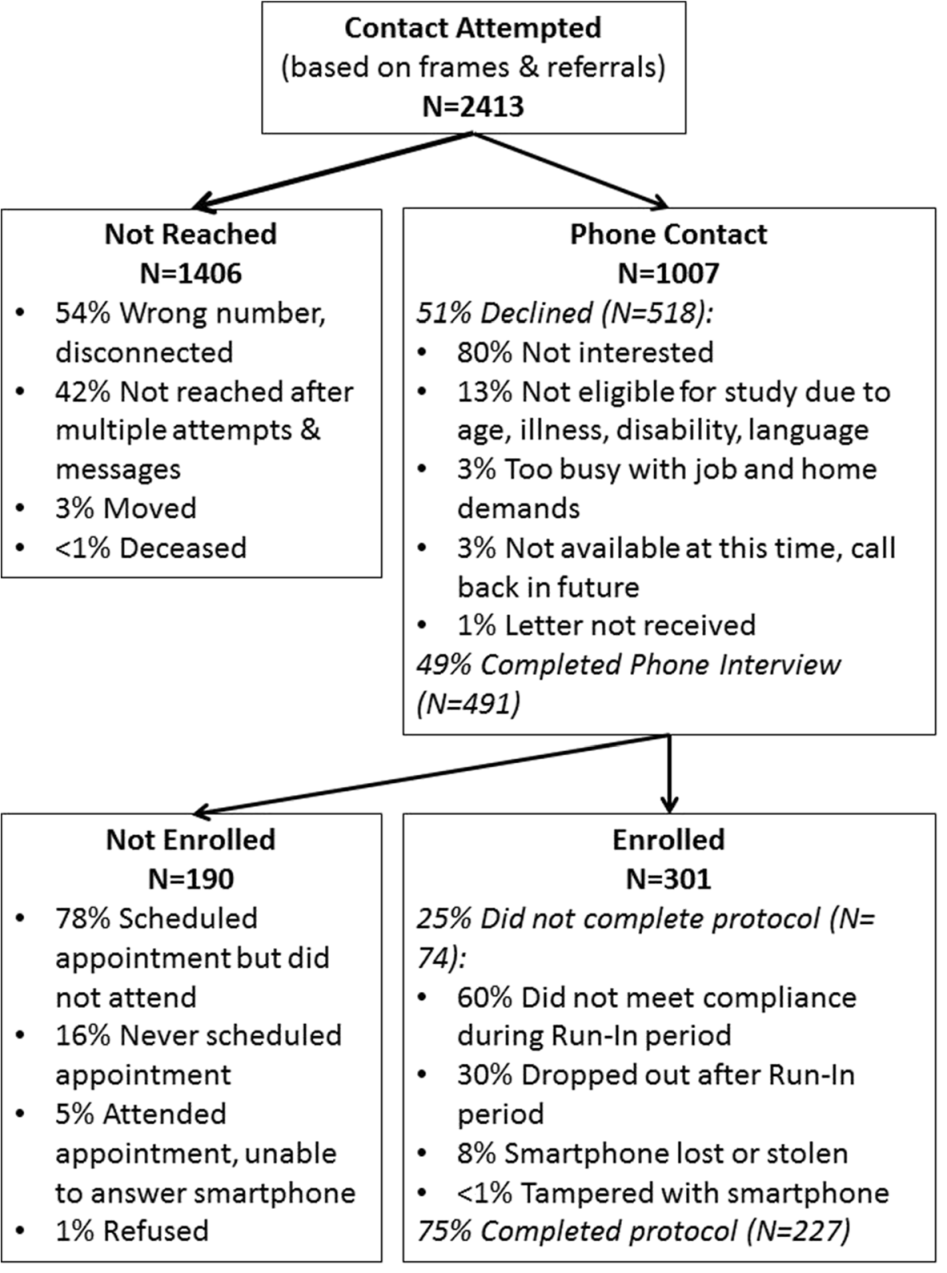
Project ESCAPE Recruitment, Enrollment, and Measurement Burst 1 Completion as of Dec. 2014

#### Physiological measures of stress and endocrine function

Our primary marker of endocrine function in ESCAPE is cortisol, which is the primary glucocorticoid (GC) in humans and an important stress hormone. The majority of our cortisol variables were derived from salivary assessments, including cortisol awakening response (CAR) and area under the curve (AUC). The CAR is of particular importance to the ESCAPE study because it can be used to identify a blunted CAR, which is a signature of chronic stress [48]. Blood samples were also used to determine plasma cortisol and dehydroepiandrosterone sulfate (DHEAS). DHEAS is an adrenal hormone that both suppresses cortisol production and antagonizes its immunosuppressive (anti-inflammatory) effects [49, 50]. As a low DHEAS/cortisol ratio has been linked with a range of clinical outcomes, including cognitive impairment and senile dementia [51], the ESCAPE study will evaluate the effects of stress on this ratio and its role in explaining the impact of stress on cognition. Further, DHEAS declines with age and reductions in the DHEAS/cortisol ratio may explain why the deleterious consequences of HPA axis activation increase with age [52–56].

#### Assay details

At the end of each burst, cortisol levels were determined employing a competitive solid phase time-resolved fluorescence immunoassay with fluorometric end point detection (DELFIA). The intra-assay coefficient of variation (CV) is 4.0-6.7 % and the inter-assay CV is 7.1-9.0 %. Plasma, obtained at the end of each burst, is assayed to determine cortisol and DHEAS levels by enzyme-linked immunosorbent assay (ELISA) (IBL International Corp., Toronto ON). The minimum detection limit for cortisol is 2.46 ng/ml, and its inter- and intra-assay CVs are 2-5 %. The minimum detection limit for DHEAS is 4 pg/ml and its inter- and intra-assay CVs are 4-7 %. All of the above assays were performed in duplicate.

#### Physiological measures of inflammation

The majority of studies linking cognition and inflammation have not examined more than one or two inflammatory markers at a time. This study takes a multivariable approach, and simultaneously assesses ten pro- and anti-inflammatory biomarkers: interleukin (IL)-1β, IL-2, IL-4, IL-5, IL-6, IL-8, IL-10, tumor necrosis factor (TNF)-α, interferon (IFN)-γ and granulocyte macrophage colony-stimulating factor (GM-CSF; assay details described below). We also determined circulating plasma levels of C-reactive protein (CRP), which has widespread use clinically as a well-validated and sensitive marker of systemic inflammation [57]. Stimulated *ex vivo* inflammatory cytokine responses were also measured. For this purpose, a subset of whole blood (1 mL) was incubated with 1 μg/mL lipopolysaccharide (LPS). LPS is an antigen that stimulates immune cells and can thus be used to quantify the inflammatory response that cells in whole blood generate to an immune challenge. This provides a dynamic measure of inflammatory change that is unique from that of basal inflammation, and which may differentially relate to stress and cognition. Because not all cytokine levels rise significantly in response to *ex vivo* LPS stimulation, a subset of cytokines were assessed in LPS-stimulated blood: IL-1β, IL-6, IL-8, IL-10, TNF-α, and GM-CSF.

Importantly, the various inflammatory biomarkers determined in the ESCAPE study serve different functions and may therefore exhibit different response patterns to stress. This study thus allows for an inflammatory profile to be determined for each individual at a given time point, which is more reliable, valid, and informative than the rise or fall of a single biomarker. We expect the pattern in which these profiles change across years will be more predictive of cognitive aging than changes in any single measure of inflammation.

#### Assay details

Basal and LPS-stimulated cytokine levels were determined using multiplex magnetic bead arrays (Life Technologies, Grand Island NY). Multiplex bead arrays have advantages over standard ELISAs, including simultaneous measurement of interrelated cytokines, high sensitivity, and reductions in inter-plate variability, cost of analysis, and the amount of time and sample needed. The minimum detection limit for these assays is less than 0.5 pg/ml for each analyte and inter-assay CVs are 4.4 %-8.6 %. CRP is determined from blood plasma using ELISA (Cayman Chemical, Ann Arbor MI); the minimum detection limit is .047 ng/ml (.000047 mg/L) and intra-assay CV is 2 %-7 %. All assays were performed in duplicate.

#### Physical and mental health

ESCAPE includes both objective and subjective assessments of health. In order to examine longitudinal changes in indicators of overall *physical health*, participants completed blood draws and physical assessments (e.g., BP, waist-to-hip ratio). Participants also provided subjective reports of their physical functioning, medical history, and medication use.

Overall *mental health* was assessed via self-reports of anxiety, depression, and anger using standardized measures from the National Institutes of Health (NIH) Patient Reported Outcomes Measurement Information System (PROMIS). In the momentary smartphone surveys, participants reported on their current emotional states (anger, anxiety, depression), which will allow us to track predictors of dynamic shifts in affective states as well as obtain more ecologically valid assessments of mental health than single-shot questionnaires can provide.

#### Risk and protective factors

Individual difference characteristics (i.e., personality, psychosocial resources, life orientation) as well in as lifetime experiences (i.e., childhood adversity, positive life events) may moderate the relationships among stress, URT, and cognitive function. ESCAPE includes measures of both risk and protective factors, as described in Table 1.

### Analytic approach

As described above, the primary objectives of the ESCAPE study are to examine whether URT operates as an important psychological pathway by which stressors exert their adverse effects on cognitive health, and to examine whether stress-related alterations in HPA-axis function and inflammation help explain those connections at a physiological level. These objectives will be accomplished by examining both the short-term effects and long-term effects of stress on cognitive function.

#### Short-term tests

With regard to shorter term effects, we will evaluate the hypothesis that URT contributes to short-term (daily) stress effects on cognition via two sets of analyses. First, we will fit an autoregressive multilevel mixed model (MLM; [58]) to burst data to establish a lagged association between daily stress and ambulatory cognitive function, controlling for current stress, fatigue, mood, and other momentary covariates. Multilevel modeling is required because the burst data consists of repeated observations nested within persons. After establishing the lagged effect of daily stressors on working memory, we will then follow multi-level mediation procedures described in Bauer and colleagues [59] to test whether daily URT significantly mediates this relationship. Second, we will use structural MLM [60] to examine whether components of daily stress (e.g., exposure and reactivity) predict higher levels of systemic inflammation and altered inflammatory responses, and whether inflammation explains the association between daily stress and ambulatory memory function.

#### Long-term tests

To elucidate longer-term effects, we will use structural equation modeling (SEM; [61]). In these models, we will test the hypotheses that URT (at the psychological level), and evidence of HPA-axis dysregulation and inflammation (at the physiological level), mediate long-term effects of chronic stress on cognitive function. We will use baseline data to first determine whether individual differences in the tendency to engage in URT mediates the effects of chronic stress on current levels of ambulatory (assessed by smartphone) and lab-based measures of cognitive function. These analyses will be followed by SEM analyses that examine whether 1) those who have experienced greater chronic stress will have lower (blunted) values for cortisol area under the curve (AUC) and CAR, and elevated basal and inflammatory responses, 2) a blunted AUC and CAR predict greater inflammation, and 3) inflammation account for associations between chronic stress and cognition measures. Next, we will conduct longitudinal analyses using MLM to test the prediction that URT mediates the effects of chronic stress on the rates of cognitive change across measurement bursts. In this analysis, chronic stress will be treated as a time-varying predictor of intraindividual cognitive change across the four year follow-up, and URT will be examined as a time-varying mediator. Similarly, in analyses adding in HPA-axis and inflammatory markers we expect that AUC, CAR, and inflammatory changes will mediate the effects of chronic stress on intraindividual cognitive change. Sliwinski and colleagues provide a detailed description and example of this analytic approach in a study that examined long-term intraindividual changes in daily stress and negative affect [62].

#### Missing data/attrition

Some participants will not complete all planned measurements. Our use of general linear mixed models will insure unbiased results as long as missing data depend only upon observed variable (i.e., missing at random [MAR]). We will conduct sensitivity analysis using pattern mixture methods (e.g., [63]) to assess possible effects of informative attrition on study conclusions. Item-level missing data will be imputed using standardized guidelines specific to each questionnaire when available (e.g., PROMIS measures). In other cases, if the amount of missing data for a questionnaire is small (e.g. <5 %), we will consider calculating prorated scale scores to minimize casewise deletion.

#### Sample size calculations

Our power analysis considered sample size requirements for conducting both between-person and within-person statistical tests with an alpha level set to .05. For testing between-person effects we determine the necessary sample for detecting a small effect of stress on cognition (partial r = .16) using a general linear model. We selected this effect size based on our previous work [14]. This yielded a necessary sample size of approximately N = 310. Determining power for within-person tests conducted by multilevel analysis is complex, as values for many different parameters (e.g., intercept, slope, residual variance, covariances) must be specified. For simplicity, we assumed dependent variables were standardized (mean = 0, variance = 0) and the intraclass correlation ranged between .4 and .6 for the smartphone cognitive measures. Using these assumptions we conducted Monte Carlo (using SAS IML and MPLUS) simulations to determine the minimum detectable within-person effect assuming an initial sample of 300 and attrition rate of 20 % at burst 2, and 10 % at each burst thereafter. Results from our simulations yielded power of at least .80 for detecting small within-person effects on cognition that could account for at least 3 % of the variance.

## Discussion

ESCAPE takes a multi-pronged approach to assessing stress (i.e., early adversity, chronic strains, major events, daily hassles), psychological mediators (e.g., URT), biological mechanisms (i.e., HPA function, inflammation) and cognitive outcomes across different time-scales (i.e., momentary ambulatory cognitive performance, decline across years). In addition to its basis in theory and use of a longitudinal intensive repeated measures design, the implications for this study are strengthened by the use of systematic probability sampling which allows comparison with national norms on key markers of well-being. To date, 301 participants have been enrolled in ESCAPE, with 227 qualifying for and completing the first burst protocol thus far (see Fig. 3 for a diagram of participant recruitment, enrollment, and compliance to date). Data collection and analysis are ongoing. Despite the intensive nature of the protocol, participants completed a median of 83 % of all smartphone surveys and ambulatory cognitive tests. A majority of participants are female (66 %), Black/African-American (58 %), and have at least a high school degree (78 %), and the sample has a median income of approximately $49,000. See Table 2 for complete demographics. The measures selected allow for the comparison of the ESCAPE sample with national norms on physical and psychological functioning. The average self-reported mobility and ability to perform instrumental activities of daily living in the ESCAPE sample (50.9 [range: 23.4 – 61.7]) were similar to national norms on the relevant PROMIS measures, which have a mean of 50 and standard deviation of 10. On average, levels of emotional distress in the ESCAPE sample (Anger: 53.2 [range: 32.4 – 76.7], Depression: 53.1 [range: 38.2 – 81.3]; [64]) are modestly higher than PROMIS national norms, with levels of anxiety (56.3 [range: 36.3 – 75.8]) nearly half a standard deviation above U.S. averages. By also including this comparison with national norms, the results of future studies of stress, emotion, physiological dysregulation, and cognitive performance can be more objectively compared.

**Table 2:** Demographics

Mean age in years (SD)	47.35 (10.99)
Sex (%)	
Male	36.30
Female	63.70
Ethnicity/Race (%)	
Non-Hispanic white	8.16
Black	59.18
Hispanic, White	20.07
Hispanic, Black	8.16
Other	4.42
Income (%)	
Less than 4999	5.86
5,000-19,999	15.52
20,000-39,999	23.79
40,000-59,999	20
60,000-79,999	12.76
80,000-99,999	6.9
100,000-149,999	6.9
150,000 or more	1.38
Chose not to answer	6.9
Marital Status (%)	
Married to first spouse	23.44
Remarried	10.26
Divorced	12.82
Separated	6.23
Never married	35.16
Widowed	2.93
Not married but living with someone	9.16

### Significance of project ESCAPE

There are currently very few empirical studies that prospectively link environmental, psychological and biological facets of the stress process to cognitive outcomes in the same sample. Specifically, despite the body of research that has established pairwise associations between stress-related variables and cognitive function, there has been relatively little research that directly examines the explanatory power of candidate mediators of stress effects on cognitive function, Project ESCAPE will address this gap in our understanding of how stress impacts cognitive function through innovative combination of data collected at multiple time scales and multiple theorized pathways. First, the ambulatory data collection via smartphones enhances ecological validity and the intensive repeated measurements will help establish causal ordering of the effects of everyday stressors on thoughts, mood, and cognitive performance. For example, measures of daily stress are related to poorer cognitive performance [9], and high levels of URT are associated with lower cognitive performance [e.g., 10, 63]. However, no study to date has evaluated any mediational hypotheses regarding the short-term effects of daily stress on cognition. The present project will be able to differentiate the momentary effects of daily stress on ambulatory cognition into components associated with attention depletion (indexed by URT) from the influence of other stress-related effects (e.g., mood, fatigue).

Second, the longitudinal data collected across four years will allow us to examine relationships between cumulative and long-term stressors, URT, dysregulations of the HPA axis and inflammation, and cognitive decline. Some studies have shown that chronic stress is related to dysregulated HPA-axis function and elevated systemic inflammation [19, 20, 66]. Other studies have shown that HPA-axis dysregulation is linked with poorer cognitive function [23, 67, 68] and still others have identified a relationship between systemic inflammation and cognitive outcomes [69, 70]. None of these studies, however, have examined prospective associations among stress, HPA-axis function, inflammation, and cognition in the same sample, nor done so in a longitudinal fashion. ESCAPE is designed to jointly test these relationships and to evaluate URT and physiological dysregulation as mechanisms explaining how stress affects cognitive function on a daily basis as well as over the long-term.

ESCAPE is positioned to improve our understanding of how environmental, psychological and physiological stress-related influences accumulate and interact to affect cognitive health. The concurrent assessment of psychosocial, physiological, and cognitive variables will permit us to model pathways by which stress can lead to cognitive decline in middle-aged adults. By measuring these facets in a concurrent fashion across a broad time scale ranging from moments to years, ESCAPE will yield fine-grained data to advance our understanding of the underlying mechanisms by which stress affects cognitive health, and will inform intervention strategies that target these mechanisms.
